# A systems biology approach to identify effective cocktail drugs

**DOI:** 10.1186/1752-0509-4-S2-S7

**Published:** 2010-09-13

**Authors:** Zikai Wu, Xing-Ming Zhao, Luonan Chen

**Affiliations:** 1 Institute of Systems Biology, Shanghai University, Shanghai, China; 2 Business School, University of Shanghai for Science and Technology, Shanghai, China; 3 School of Communication and Information Engineering, Shanghai University, Shanghai, China; 4 Key Laboratory of Systems Biology, SIBS-Novo Nordisk Translational Research Centre for PreDiabetes, Shanghai Institutes for Biological Sciences, Chinese Academy of Sciences, Shanghai, China; 5 Computational Biology Research Center, National Institute of Advanced Industrial Science and Technology, Tokyo, Japan

## Abstract

**Background:**

Complex diseases, such as Type 2 Diabetes, are generally caused by multiple factors, which hamper effective drug discovery. To combat these diseases, combination regimens or combination drugs provide an alternative way, and are becoming the standard of treatment for complex diseases. However, most of existing combination drugs are developed based on clinical experience or test-and-trial strategy, which are not only time consuming but also expensive.

**Results:**

In this paper, we presented a novel network-based systems biology approach to identify effective drug combinations by exploiting high throughput data. We assumed that a subnetwork or pathway will be affected in the networked cellular system after a drug is administrated. Therefore, the affected subnetwork can be used to assess the drug's overall effect, and thereby help to identify effective drug combinations by comparing the subnetworks affected by individual drugs with that by the combination drug. In this work, we first constructed a molecular interaction network by integrating protein interactions, protein-DNA interactions, and signaling pathways. A new model was then developed to detect subnetworks affected by drugs. Furthermore, we proposed a new score to evaluate the overall effect of one drug by taking into account both efficacy and side-effects. As a pilot study we applied the proposed method to identify effective combinations of drugs used to treat Type 2 Diabetes. Our method detected the combination of Metformin and Rosiglitazone, which is actually Avandamet, a drug that has been successfully used to treat Type 2 Diabetes.

**Conclusions:**

The results on real biological data demonstrate the effectiveness and efficiency of the proposed method, which can not only detect effective cocktail combination of drugs in an accurate manner but also significantly reduce expensive and tedious trial-and-error experiments.

## Background

A cellular system is a complex molecular network in which proteins, DNAs, metabolites and other small molecules interact with each other, so that the cellular machine functions properly [1]. A subsystem or even the whole system will be affected if one or several components are influenced by genetic or epigenetic factors, which is manifested in diseases. In general, many diseases result from multiple genetic and environment factors, and cause severe dysfunction of some cellular components, which can be exemplified by cancer and Type 2 Diabetes. To restore the individuals with such diseases, agents that can intervene different disease factors simultaneously are highly demanded. In the past decades, one-target one-drug paradigm has been the dominating drug discovery approach [2,3], which leads to many drugs marketed but cannot treat certain complex diseases sufficiently. Furthermore, if such a drug is administered over a long time, some mutations in patients may take place and therefore trigger drug resistance. On the other hand, the possibility of developing drug resistance will be reduced greatly if several drugs are administered simultaneously, and such an effect can be seen in the cocktail treatment for HIV infection. In other words, it is possible to discover new drugs if the marketed drugs are combined in an appropriate way. Since the combination drugs are based on approved single drugs, the combination drugs will be safe with less adverse reactions and have shown promising potential for new drug discovery. In fact, it has been a long history to use combination drugs for treating diseases and reducing suffering. For example, the Traditional Chinese Medicines (TCM), especially herbal medicines, which can be viewed as the combinations of multiple compounds with synergy effects, have been used for thousands of years [4]. Recently, with the development of medicine science and pharmacology industry, combination drug is becoming the standard of care for many complex diseases. As a result, some methods have been proposed to identify effective drug combinations. These methods can be grouped into two classes, i.e. computation based methods and experiment based methods.

When a drug with different dosage is administered, an organism may respond differently and therefore the efficacy of this drug may manifest differently. The dose-response relationship is generally represented as a dose-response curve, in which response is usually measured as the percentage of the inhibited cell's growth rate or the percentage of cells that are killed by the drug. Dose-response curve is the common basis for computation based methods to identify effective drug combinations. With dose-response curve in hand, one can define the null model to describe the relationship between dose and response of the combination drug whose members have no interaction. Finally, based on the comparison between the predicted dose-response curve of the null model and a real dose-response curve of combination drugs, synergism, additive and antagonism between drugs can be defined accordingly. For instance, under the assumption that two inhibitors acting on a target through similar mechanisms, Loewe proposed an additivity model to predict the combined effect of two inhibitors [5]. In this model, the combined effect of two inhibitors are defined as an implicit function [*CI*_1_]/[*I*_1_] + [*CI*_2_]/[*I*_2_], where [*I*_1_] and [*I*_2_] are the concentrations of drugs 1 and 2 respectively, with which drug 1 or 2 alone can inhibit a target by a specified percentage. [*CI*_1_] and [*CI*_2_] are the amount of inhibitors 1 and 2 used in the combination drug which inhibits target activity as the same as single inhibitors 1 and 2. Based on this function, synergy, additive and antagonism are defined respectively, where [*CI*_1_]/[*I*_1_] + [*CI*_2_]/[*I*_2_] > 1 implies synergy, [*CI*_1_]/[*I*_1_] + [*CI*_2_]/[*I*_2_] = 1 implies additive effect, and [*CI*_1_]/[*I*_1_] + [*CI*_2_]/[*I*_2_] < 1 corresponds to antagonism [6]. By assuming that two inhibitors act through independent mechanisms, Bliss proposed another null model to define combined effect of two inhibitors [7]. In this model, the combined effect of two inhibitors is predicted as the multiplication of single inhibitors's effect and represented as the union of two probabilistically independent events [6]. Recently, many researchers have devoted themselves to extend these two models to computationally search effective drug combinations [6]. Based on mass action law, Chou and Talalay unified all existing models and proposed a general model to define combined effect of multiple drugs [4]. In the model, a median-effect equation was defined to describe dose-effect relationship and a Combination Index (CI), i.e. , was proposed to quantify synergism or antagonism. In the formula, (D*_x_*)*_j_* is the dose with which the j-th drug “alone” can inhibit a system by x% and ((*D*)_1_, …, (*D*)*_*n*_*) is the dose vector with which drug combination can inhibit a system by x% .

On the other hand, some important combination drugs have been discovered by experiments. For example, Agrawal et al. found a combination drug for treating Huntington disease based on experiments in Drosophila [8]. Since combination drug is becoming the standard of care, there are many papers describing clinical rules about how to combine 2-3 drugs [9]. These rules are all from clinical experience or randomized clinical trials and have been used clinically to treat cancer, Type 2 Diabetes, bacterial, Huntington disease, and so on [10]. Compared with the computation based methods, these experiment based methods are more reliable and can be applied to treat patients more directly. But the combination drugs identified by this kind of methods are only composed of 2-3 frequently used drugs due to the limitation of experiment resources, thereby limiting the space of possible combination drugs and missing many potential combination drugs. Under the circumstance, a high-throughput screening method was recently proposed to identify effective combinations of therapeutic compounds [11]. In [10,12], two stochastic search algorithms were developed to identify effective combinations of drugs. In these two methods, biological response information such as the percentage of cancerous cells being killed was utilized to detect an appropriate solution, i.e. proper dose of each drug. In [13], based on the data quantifying the the effect of single drugs over individual strains, a minimal hitting set based method was proposed to identify drug combinations that can have putative effective response to heterogeneous population of malignant agents.

Although above mentioned methods identified many effective drug combinations, there still exists much room to be improved. For example, the methods mentioned above except clinical experience based methods did not consider side effect of drugs explicitly or sufficiently while searching or evaluating drug combinations. A good drug combination should have less side effect but more efficacy. Another limitation of existing methods is that they are blackbox-like methods to some extent, and thereby makes it difficult to explain why the drugs work. That is, it is largely unknown why the identified combination of drugs works while other combinations not.

Inspired by rapid progress of high throughput technology and recent research works about systems biology, we proposed a novel network-based method to identify effective drug combinations based on gene expression data of individual drugs. In particular, we constructed a background molecular interaction network, and predict gene expression profile of one combination drug based on microarray data of individual drugs with a new computational scheme. Furthermore, we developed a new integer programming model to identify pathways or subnetworks affected by single drugs or combination drug from the background network by exploiting gene expression data. Moreover, we designed a score by taking into account efficacy and side-effect based on the identified subnetworks affected by drugs, and quantitatively evaluate all possible combinations of drugs and identify the best candidates of combination drugs. As a pilot study, we applied the method to identify effective combinations of drugs used to treat Type 2 Diabetes. The results on real biological data demonstrate the effectiveness and efficiency of the proposed method, which can not only detect effective cocktail combination of drugs in an accurate manner but also significantly reduce expensive and tedious trial-and-error experiments. In addition, the proposed approach can be used to computationally predict the gene expression profiles generated under multiple perturbations based on the gene expression profiles by individual perturbations.

## Results and discussions

In this part, we applied our method to Type 2 Diabetes mellitus, which is one of leading complex diseases that threat the health of human beings worldwide [14]. It is defined by the abnormal high blood glucose level which causes profound metabolic dysfunction. Although some drugs have been approved by American Food and Drug Administration(FDA) to cure it, most of them have only single target and cannot cure this complex disease effectively and sufficiently. In this paper, we applied our method to identify effective drug combinations for treating Type 2 Diabetes.

In the data set used here, there are six approved drugs for Type 2 Diabetes with available microarray data, i.e. Metformin, Phenformin, Chlorpropamide, Tolbutamide, Rosiglitazone, and Troglitazone. Since two targets of Troglitazone are not included in the background network, we use the other five drugs, i.e. Metformin, Phenformin, Chlorpropamide, Tolbutamide and Rosiglitazone for the drug combination prediction and test. Metformin was approved in 1994 to treat Type 2 Diabetes. It works by decreasing hepatic gluconeogenesis. Rosiglitazone was approved in 1999. It works by improving insulin sensitivity. Several years ago, the combination of Metformin and Rosiglitazone, i.e. Avandamet, was approved to treat Type 2 Diabetes. Compared with Metformin or Rosiglitazone clinically, Avandamet can improve glycemic control, insulin sensitivity without new tolerability issues in some populations under certain circumstances [15-19]. In this paper, we intend to elucidate the mechanism underlying Avandamet and investigate why it works better than Rosiglitazone or Metformin, based on the proposed computational method. In addition, we also aim to identify other potential effective drug combinations based on our method.

### Prediction of effective drug combinations for Type 2 diabetes

There are some Type 2 diabetes related genes that have been identified by Genome Association Analysis, such as those deposited in OMIM database [20,21]. On the other hand, several methods have been proposed to predict disease related genes based on differential gene expression information. In [22], six computational methods were integrated to predict Type 2 diabetes related genes. To assess the efficacy of drugs, we used the genes predicted by at least five methods for Type 2 Diabetes from the supporting information of [22]. Subsequently, the list of Type 2 diabetes related genes obtained from OMIM database and the list from [22] were merged. Of the genes in the merged list, 54 genes exist in our background network. These 54 genes constitute the reference set of Type 2 Diabetes related genes. On the other hand, to assess the side-effect of drugs, we used the essential genes which are defined to be the orthologs of essential genes found in mouse, and the list of essential genes in the mouse were obtained from MGI (http://www.informatics.jax.org/).

**Figure 1:**
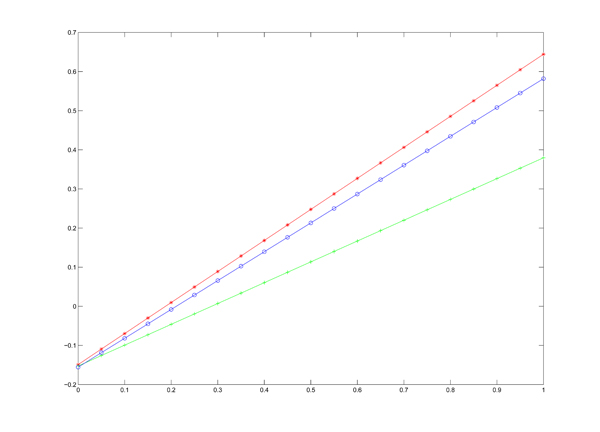
**The scores of three subnetworks** The figure gives the scores of subnetwork affected by Avandamet, Metformin, and Rosiglitazone with different parameter λ, where the x-axis and y-axis denote the value of λ and score respectively.

Since there are no gene expression data treated with drug combinations, gene expression profiles caused by them were predicted based on gene expression data treated with single drugs as described in section 5.2. Subsequently, the subnetworks affected by single drugs and the drug combinations were identified. The five single drugs can make up ten possible 2th-order combinations, that is, Rosiglitzone& Tolbutamide, Rosiglitazone&Chlorpropamide, Rosiglitazone&Phenformin, Tolbutamide&Chlorpropamide, Tolbutamide&Chlorpropamide, Tolbutamide& Phenformin, Tolbutamide&Metformin, Chlorpropamide&Phenformin, Chlorpropamide&Metformin, Phenformin&Metformin, and Metformin&Rosiglitazone. We used the proposed method to identify which of them are effective drug combinations. As shown in Table 1, according to the comparison of scores of subnetworks affected by drug combinations over those of single drugs, Rosiglitazone&Chlorpropamide and Metformin&Rosiglitazone were identified as effective drug combinations, which have higher scores than those of individual drugs. Actually, Metformin&Rosiglitazone is Avandamet, which is an approved combination drug, thereby verifying the effectiveness of the proposed method. Figure 1 shows the scores of Avandamet, Metformin and Rosiglitazone in detail for different λ. In addition, the prediction for the combination of Rosiglitazone&Chlorpropamide is also reasonable because Rosiglitazone and Chlorpropamide works by different mechanisms. Rosiglitazone works by increasing insulin action while Chlorpropamide works by increasing insulin secretion. Therefore, it may work better when they are used in combination. Of course, this prediction needs to be further verified clinically in experiment on populations. On the other hand, the other eight combinations were predicted as ineffective drug combinations. Their ineffectiveness also need verified clinically or experimentally.

**Table 1:** Prediction of drug combinations

Combination Drug	Score of Combination Drug	Scores of Individual Drugs	Prediction Results
Ros&Met	0.247611	(0.213158, 0.113546)	+
Ros&Chl	0.219065	(0.213158, 0.173858)	+

Ros&Tol	0.158606	(0.213158, 0.014646)	_
Ros&Phe	0.158969	(0.213158, 0.163439)	_
Tol&Chl	0.084770	(0.014646, 0.173868)	_
Tol&Phe	0.082675	(0.014646, 0.173868)	_
Tol&Met	0.084520	(0.014646, 0.163439)	_
Chl&Phe	0.161848	(0.173868, 0.163439)	_
Chl&Met	0.145706	(0.173868, 0.113546)	_
Phe&Met	0.117798	(0.163439, 0.113546)	_

Prediction of drug combinations, in which Ros, Tol, Chl, Phe and Met are the abbreviations of Rosiglitazone, Tolbutamide, Chlopropamide, Phenformin and Metformin respectively. + and _ denote effective combination and noneffective combination respectively. The scores listed in the table were obtained by setting λ to 0.5.

**Figure 2:**
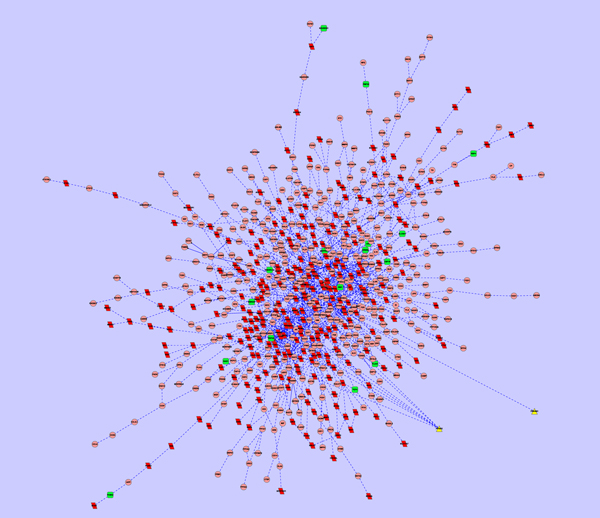
**Subnetwork affected by Avandamet**, where nodes with triangle shape, nodes with green color, and nodes with red color denote drug target, disease gene, and essential gene, respectively.

**Figure 3:**
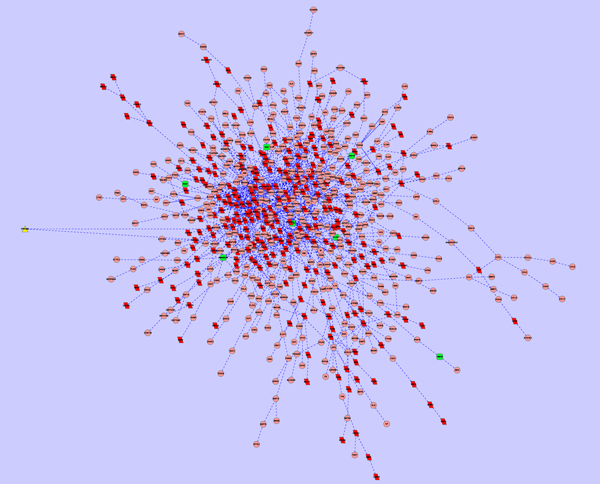
**Subnetwork affected by Metformin**, where nodes with triangle shape, nodes with green color, and nodes with red color denote drug target, disease gene, and essential gene, respectively.

**Figure 4:**
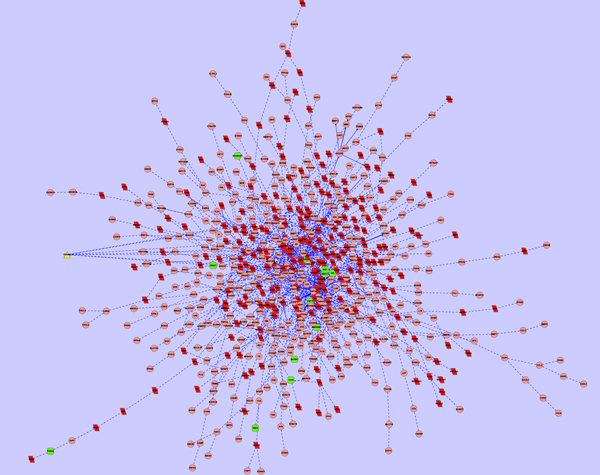
**Subnetwork affected by Rosiglitazone**, where nodes with triangle shape, nodes with green color, and nodes with red color denote drug target, disease gene, and essential gene, respectively.

As a demonstrated example, subnetworks affected by Avandamet, Metformin and Rosiglitazone were shown in Figure 2, Figure 3, and Figure 4. In the subnetwork affected by Avandamet (combination drug), 16 Type 2 Diabetes related genes are included, and are listed in Table 2. Table 2 also lists 7 Type 2 Diabetes related genes affected by Metformin, and 12 such genes affected by Rosiglitazone. With close examination of Table 2, we found that most of the disease related genes in the subnetworks affected by Metformin or Rosiglitazone are also in the subnetwork affected by Avandamet, which explains why Avandamet (combination drug) outperforms Rosiglitazone or Metformin to some extent. On the other hand, the number of essential innocent genes in the three identified subnetworks are 271, 266, and 242 respectively, which explain why Avandamet will not introduce new tolerability issues to some extent. To quantitatively measure the advantage of Avandamet over Rosiglitazone or Metformin based on the identified subnetworks, the three subnetworks were evaluated by scheme (14). It is worth noting that scheme (14) is the function of parameter λ, which was introduced to balance the measure for efficacy and side effect. Therefore, we changed λ from zero to one while investigating scores of the three identified subnetworks. When λ was varied from zero to one by 0.05, the objective value for given λ was recorded. Fig. 1 gives scores of three subnetworks corresponding to three drugs when increasing the parameter λ from zero to one. It can be seen from the figure that scores of Avandamet are always higher than that of Rosiglitazone or Metformin regardless of the parameter λ, which agrees with the clinical conclusion very well. The results show that our method can successfully identify effective combination drug, i.e. Avandamet, which demonstrates the efficiency of the proposed method and also proves the necessity to understand working mechanism of drugs from perspectives of systems biology.

**Table 2:** Type 2 Diabetes related genes affected

Gene symbol	Corresponding protein name	Ros&Met	Met	Ros
*TCF4*	Transcription factor 7-like 2	Yes	No	Yes
*MAPK8IP1*	C-jun-amino-terminal kinase-interacting protein 1	Yes	No	Yes
*NEUROD1*	Neurogenic differentiation factor 1	Yes	No	Yes
*HNF4A*	Hepatocyte nuclear factor 4-alpha	Yes	No	No
*IRS1*	Insulin receptor substrate 1	Yes	No	Yes
*IRS2*	Insulin receptor substrate 2	No	Yes	No
*AKT2*	RAC-beta serine/threonine-protein kinase	No	Yes	No

*AXL*	Protein AXL2	Yes	No	Yes
*ERBB2*	Receptor tyrosine-protein kinase erbB-2	Yes	No	Yes
*PCSK2*	Neuroendocrine convertase 2	Yes	No	Yes
*RBP4*	Retinol-binding protein 4	Yes	No	Yes
*SLC8A1*	Sodium/calcium exchanger 1	Yes	No	Yes
*IKBKAP*	Elongator complex protein 1	Yes	No	No
*SMARCA4*	Probable global transcription activator SNF2L4	Yes	Yes	No
*PMP22*	Peripheral myelin protein 22	Yes	Yes	No
*CSF1R*	Macrophage colony-stimulating factor 1 receptor	Yes	Yes	Yes
*RAG1*	V(D)J recombination-activating protein 1	Yes	Yes	Yes
*PLCE1*	1-phosphatidylinositol-4,5-bisphosphate phosphodiesterase epsilon-1	Yes	Yes	No
*SHC1*	SHC-transforming protein 1	No	No	Yes

Type 2 Diabetes related genes affected by Ros&Met, Rosiglitazone and Metformin.

### Assessment of the predicted drug combinations for biological relevance

The prediction of drug combinations in this paper is mainly based on the affected subnetworks of drugs. It is difficult to assess biological relevance of drug-affected subnetworks since there is no objective criterion or gold standard to define it. Here, we performed functional enrichment analysis to evaluate biological relevance of subnetworks affected by drugs empirically. As an example, the GO term [23] and KEGG pathway [24] enrichment analysis were performed with hypergeometric test on genes involved in subnetworks affected by Avandamet, Metformin, and Rosiglitazone respectively through DAVID online web server [25], where the whole genome was used as background. The full list of enriched GO terms and KEGG pathways on the three subnetworks can be found in Additional file 1, 2, 3, 4, 5, 6. Since there are many overlaps for enriched terms and pathways among the three subnetworks, we only list the representative GO terms and KEGG pathways enriched in the subnetwork affected by Avandamet in Tables 3 and 4 respectively. It can be seen that the enriched GO terms can be grouped into four categories. The first class is composed of GO terms about response to stimulus, which is reasonable since the drug behaves as stimulus when it is administered. The second class of enriched GO terms involve many diverse and fundamental biological processes, such as transcription, metabolic, and so on. This gives hints that the drug may have comprehensive effect on the cellular system. However, the exact mechanism is still not clear and needs to be elucidated by biologist in future. Furthermore, some well known signaling pathways constitute the third class of terms. This is reasonable to consider that Type 2 Diabetes has close relations with signaling. Therefore, interfering such signaling events may contribute to the cure of disease when drugs are administered. For example, Wnt signaling pathway is enriched in the subnetwork. A resent research shows that Wnt signalling pathway that is involved in normal pancreatic development is closely related to Type 2 diabetes [26]. The enrichment of these signaling pathways demonstrates the biological relevance of the identified subnetwork and effectiveness of our method to some extent. The last class of enriched terms involve biological processes about heart contraction and blood vessel. The toxicity description of Rosiglitazone in the Drugbank database [27,28] (http://www.drugbank.ca/drugs/DB00412) is that its side effects include fluid retention, congestive heart failure (CHF), liver disease. With further examination, we found that 13 genes in the subnetwork affected by Avandamet are related to heart contraction, where six genes are essential genes. At the same time, 11 genes in the subnetwork affected by Rosiglitazone are related to heart contraction, where six genes are essential genes. Therefore, the enriched terms about heart contraction can be linked to the side effect of congestive heart failure (CHF) of Rosiglitazone and the active component of Rosiglitazone in Avandamet. In future, the reason underlying the enrichment of heart contraction related terms in the subnetwork affected by Metformin needs to be exploited in clinic. As for the enriched KEGG pathways, they can be further grouped into two classes. Not surprisingly, signaling pathways, such as insulin signaling pathway constitute the first classes. Taken together, the GO term and KEGG pathway enrichment analysis demonstrates the biological relevance of identified subnetworks and the effectiveness of our method.

**Table 3:** Representative enriched GO terms

ID	Term	P-value
0042221	response to chemical stimulus	2.74E-6
0048878	chemical homeostasis	1.94E-4
0030154	cell differentiation	1.80E-34
0012501	programmed cell death	2.19E-20
0006915	apoptosis	3.95E-20
0006796	phosphate metabolic process	8.27E-19
0043687	post-translational protein modification	1.07E-18
0009893	positive regulation of metabolic process	7.68E-17
0045449	regulation of transcription	2.33E-10
0050793	regulation of developmental process	1.17E-7
0008284	positive regulation of cell proliferation	2.26E-7
0051049	regulation of transport	7.59E-5

0007243	protein kinase cascade	2.74E-18
0007169	transmembrane receptor protein tyrosine kinase signaling pathway	2.97E-18
0000165	MAPKKK cascade	1.93E-11
0016055	Wnt receptor signaling pathway	3.52E-5
0007249	I-kappaB kinase/NF-kappaB cascade	3.28E-4
0008286	insulin receptor signaling pathway	8.26E-4

002026	regulation of the force of heart contraction	2.22E-4
0001525	angiogenesis	5.32E-4
0001568	blood vessel development	0.0015
0001944	vasculature development	0.002
0048514	blood vessel morphogenesis	0.003

Representative enriched GO terms on the subnetwork affected by Avandamet.

**Table 4:** Representative enriched KEGG Pathways

ID	Term	P-value
hsa04010	MAPK signaling pathway	3.16E-11
hsa04012	ErbB signaling pathway	6.97E-9
hhsa04660	T cell receptor signaling pathway	4.33E-6
hsa04310	Wnt signaling pathway	9.34E-6
hsa04664	Fc epsilon RI signaling pathway	2.44E-4
hsa04910	Insulin signaling pathway	0.006

hsa05220	Chronic myeloid leukemia	1.57E-9
hsa05212	Pancreatic cancer	8.44E-7
hsa05215	Prostate cancer	1.59E-5
hsa05210	Colorectal cancer	3.95E-5
hsa05222	Small cell lung cancer	5.57E-5

Representative enriched KEGG Pathways on the subnetwork affected by Avandamet.

## Conclusions and discussions

Due to the complexity nature of many diseases and ever rising drug resistance, drug combination is becoming the standard of care for many complex diseases. In this paper, we presented a new method to identify effective drug combinations. Different from existing methods, the proposed method aims to identify effective drug combinations from the perspective of network or systems biology. The main idea is that subnetworks affected by the administrated drug can be used as surrogates of overall impact brought by the drug. Keeping this in mind, we can compare the efficacy & side effect of the combination drug with those of single drugs by comparing their corresponding affected subnetworks. Especially, the problem of identifying subnetworks affected by one drug including a combination drug was formulated into an integer programming model and solved by relaxing it to a linear programming model. Furthermore, we defined efficacy or side effect respectively by using the differential expression of disease genes and essential genes under study. A new score scheme that considers efficacy and side effect simultaneously was defined and used to evaluate candidate subnetworks and identify effective drug combinations. The pilot study on identifying combination of drugs used to treat Type 2 Diabetes shows that our method can successfully identify the approved combination drug, i.e. Metformin&Rosiglitazone, and a potential combination, i.e. Rosiglitazone&Chlopropamide, which demonstrates the predictive power of the proposed method. Furthermore, the results show that the subnetworks identified by our model can indeed be used as surrogates of impact brought by drug. The results also demonstrate that the proposed method complement existing methods very well. For example, our method can identify putative drug combinations quickly and provide guidelines for further clinical test. In this work, we only applied our method to Type 2 Diabetes. In the future, to verify the reasonableness and demonstrate its power extensively, we will apply our proposed method to identify possible effective drug combinations for treating other complex diseases, such as cancer. Despite the success of the proposed method, we noticed that there are still some issues that affect its performance and hamper its further application. Firstly, there are few expression data treated with combination drug available in public right now. We developed a method to predict the gene expression variation caused by a combination drug and used it to identify subnetworks affected by the combination drug. However, it is possible that there exists a gap between real data and predicted one, which will affect the performance of our method. We believe that the performance of our method will be boosted accordingly if the expression data treated with combination drug are available. Secondly, the list of disease genes is far from complete and additional disease related genes need to be found. On the other hand, the side effect in this work is defined based on those essential genes which have no relation to any diseases, whereas there are no unified definition of essential genes and the list of essential genes may be incomplete. Therefore, the proposed score based on disease genes and essential genes may be biased. Thirdly, when the method of predicting gene expression profile was used to predict expression profile caused by 3th-order or higher order combinations, expression ratio of some genes may take negative value, which needs to be modified during computation. In near future work, we will modify our method to predict gene expression profiles caused by higher order drug combinations. Fourthly, the indiction of drug usually includes rough descriptions about drug's side effect, such as headache and so on. How to find the rough set of genes corresponding to side effect and integrate this information into the subnetwork identification model will be considered in the future.

## Methods

**Figure 5:**
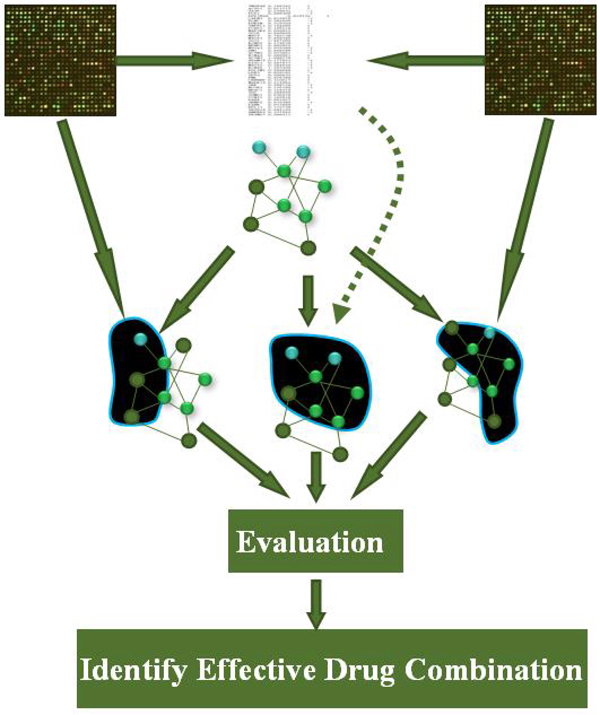
**Flowchart of our method** Flowchart to identify effective combination drugs from high-throughput data.

The idea behind the proposed method is that a subnetwork or pathway will be affected in the cellular system after a drug is administrated. Therefore, the affected subnetwork can be used to assess the drug's overall effect, and thereby help to identify effective drug combinations by comparing the subnetworks affected by individual drugs with that by combination drug. Therefore, an integer programming model is presented to detect subnetworks affected by drugs from a background molecular interaction network that is constructed by integrating protein interactions, protein-DNA interactions, and signaling pathways. Figure 5 shows the flowchart of our method. The details are addressed in the following.

### Constructing background molecular interaction network

In this paper, protein-protein interactions, protein-DNA interactions and signaling pathways were integrated and used to construct a molecular interaction network. The protein-protein interaction(PPI) data were downloaded from the HPRD database(2008, HPRD_Release_7_09012007 version), where the self-interactions and reduplicate interactions were removed. Subsequently, only interactions that were verified by at least two experimental methods were reserved. As a result, 9707 interactions were extracted which involve 4707 proteins (genes). Note that we treat gene and its product as the same hereafter. Protein-DNA interaction data were downloaded from TRED database [29], including 1273 Protein-DNA interactions in *Homo sapiens*. We also obtained signaling pathway data from the supporting information of [30]. These three data sets were integrated into one molecular interaction network that involves 5893 genes and 11726 interactions. However, only 4455 genes among these 5993 genes were expressed in the microarray data, which were involved in 7927 interactions. The 4455 genes and 7927 interactions among them form the intermediate network. Since some components do not connect with each other in the network, the biggest weakly connected component was used as the final background molecular interaction network. The background network is composed of 3644 genes with 7731 interactions.

### Predicting gene expression profiles treated with drug combination

Before assessing the effectiveness of a combination drug, in this subsection we present a new approach to predict gene expression profile treated with the combination drug based on the microarray data treated with individual drugs. The microarray data set was downloaded from the Gene Expression Omnibus (GEO) database [31,32] with the accession number GSE5258. It was originally created by Lamb et al to identify gene expression signatures that can characterize the variation caused by perturbations [33]. In the data set, many U.S. Food and drug Administration(FDA) approved drugs were used to perturb a cell line and corresponding gene expression data were obtained. At the same time, gene expression data of cell lines cultured with vehicle were used as control.

There are several microarray platforms such as GPL96 and GPL3921 were represented in the data set GSE5258. Therefore, after downloaded the raw data from GEO, the expression values of probes of each sample were transformed into expression data of genes based on the probe-gene mapping of the platform by which the sample was conducted. In detail, the average expression value of different probes corresponding to the same gene was defined as the expression value of that gene. Further, several gene symbols are pseudo, which can be exemplified by *HSPA1A /// HSPA1B*. They were formed by combining several true gene symbols. Given this, the expression value of a specified gene was set to be the mean of expression value of gene symbols that is the same as it or include it. Subsequently, batch with ID 2 and batch with ID 2a were merged into a new batch. Batches composed of samples perturbed by one single drug and their control samples were removed. To avoid the bias brought by difference in cell line used, in a batch, only samples conducted on cell line MCF7 were used further. Besides, in a batch, a case with a specified dose of drug may correspond to several controls, which makes it difficult to compare the expression change of genes in an accurate way. We define a new single control with its expression value set to the arithmetic mean of expression values of individual controls. Furthermore, one cell line may be perturbed by a specified dose of drugs several times in a batch. In this case, the gene expression data treated with the specified dose of drug were defined as the mean of expression data of individual cases, and the same for controls. Finally, if there are multiple batches that include the sample perturbed by a specified drug with specified dose, the gene expression data treated with the dose of drug were defined as the mean of expression data of individual case in each batch, and the same for controls.

Since there are no ready-to-use gene expression data treated with drug combination in the data set, we need to predict the gene expression profile caused by drug combination, based on the gene profiles treated with individual drugs. Under perturbations by the combination of several drugs, the expression value of the *i*-th gene can be represented mathematically as a function of drug doses, i.e. *f ^i ^*(*y*_1_, …, *y_n_*), where *y_j_* is the dose of the *j*-th drug. Based on Taylor expansion, if the value of *y_j_*, *j* ∈ {1, …, *n*} is near zero, *f ^i ^*(*y*_1_, …, *y_n_*) can be approximated by

	(1)

where *f ^i^* (0, …, 0) is the expression value of gene *i* without any drug perturbation (or control case) and ∂ *f ^i ^* /∂* y _j _* is the partial derivative of *f ^i^* on *y _j _*. Therefore, the ratio of expression value of the *i*-th gene perturbed with *n* drugs over that of the *i*-th gene in the control case can be represented by

 (2)

where *f ^i^* (0, …, 0) ≠ 0. For the case of individual drugs or perturbations, the ratio of expression value of the *i*-th gene perturbed only with *y_j_* = *d_j_* dose of the *j*-th drug over that in the control case can be expressed by

(3)

Substituting  of (2) with  of (3), the ratio of expression value of the *i-*th gene perturbed by *n* drugs simultaneously with new dose vector (*y*_1_, …, *y_n_*) over that without drug can be represented further by *F^i^*

It is worth noting that the above expression is based on the assumption that all experiments start with the same initial value (i.e. the same control case *f ^i^* (0, …, 0)) . However, in the biological experiments, it is not easy to ensure such conditions. For example, each case corresponds to its own control in the data set GSE5258. Therefore, we approximately represent this relation of the *i*-th gene as

	(4)

where *T* is the expression value of the *i*-th gene in the case treated with combination drug with dose vector (*y*_1_, …, *y_n_*), and *C* is the expression value of the *i*-th gene in the control. Similarly, *T_j_* and *C_j_* are the expression value of the *i*-th gene in the case treated with *d_j_* dose of the *j*-th drug and that of the corresponding control respectively. Clearly, (4) alleviates the problem with unidentical initial conditions by using the ratios.

On the other hand, the saturation or nonlinear effect should be considered when multiple drugs induce or repress the expression of the *i*-th gene simultaneously. Therefore, the representation described by equation (4) should be modified by considering such a nonlinear effect. The ratio of the *i*-th gene's expression value treated with the combination drug over that in the control is defined as follows:

With such a scheme, we can predict the gene expression profiles of multiple perturbations or a combination drug, based on the gene expression profiles of individual perturbations.

### Identifying subnetworks affected by drug

When a drug is administered, the cellular system will be perturbed, where the target molecules, e.g. genes are first affected and the effect may subsequently propagate through the networked system. As a consequence, a subnetwork connecting the target molecules will be affected significantly and lead to dysfuntion of the system. In other words, a subnetwork of genes rather than isolated genes are affected by a drug. With this in mind, we develop a new method to identify the subnetwork affected by drugs. In our work, a molecular interaction network is represented as a graph *G* = (*V*, *E*, *W*), where *V* represents the set of molecules or nodes, *E* represents the set of interactions between nodes, and *W* is the set of weights defined by the differential expression change of genes. In detail, the weight of gene *i* is defined as , where *T_i_* is the expression value of gene *i* in a case sample and *C_i_* is the one in a control sample. The subnetwork affected by drug can be seen as a subnetwork consisting of genes that differentially expressed significantly before and after drug administration. With the weighted graph generated above, the task therefore becomes into looking for an maximum-score subnetwork. In literature, there are some approaches to identifying subnetworks or pathways under specific conditions [34-42]. In this work, we introduce a new network flow model to find the maximum-score subnetwork. The network flow model was originally proposed by Lee and Dooly to model a constrained maximum-weighted connected graph that finds maximum-score subnetworks of size *R* with a fixed root node [43]. In this case, drug combinations have multiple targets, which are all needed to be included in the subnetwork to be found. Therefore, a dummy node, namely a drug node, and additional constraints are introduced into the original model. The model is formulated as follows:

(5)

(6)

(7)

(8)

(9)

(10)

(11)

(12)

(13)

where *S* denotes dummy node, *H* = {*H*_1_,*H*_2_, …, *Hk*} is the set of *K* drug targets,  is the operator of union for two sets, and |*V*| is the number of elements of *V*. Binary variable *x_i_* (*x_i_* ∊ {0,1}) denotes whether the *i*-th gene is included in the subnetwork. The number of units of flow from the *i*-th gene to the *j*-th gene is signified by *Z_ij_*. Furthermore, all the constraints introduced aim to ensure a connected subnetwork with *R* nodes. In detail, constraint (6) means that there are *R* units of flow entering the set of targets. The constraint also aims to select *R* genes for the subnetwork. The constraints (7) and (8) mean that one unit of flow will leave the network if one gene is selected. The constraint (9) is to ensure that there exists a path linking dummy node and the selected node. Finally, the objective function , which is similar to Z score, is defined to find a subnetwork with overall weight as large as possible.

Due to the NP-hard nature of this integer programming model, we relax it to a linear programming model in practice. The solution of the linear programming is obtained by an open source software glpsol.exe. Due to the relaxation, non-zero entries in variable x in solution will define the final identified subnetwork. Therefore, the size of it may be higher than *R* more or less. Theoretically, we should solve different models with *R* varies from 1 to |*V*| and find the subnetwork with the maximum score. However, one drug can just affect a subset of genes in the network by taking into account side-effect. Therefore, we assume that *R* is at most as large as 10% of the number of nodes in the network. On the other hand, to ensure the connectivity of the identified subnetwork, the lower bound of *R* is set to the number of nodes in the shortest paths between different drug targets. Finally, the subnetworks with the maximum score will be identified as the subnetwork affected by the drug.

### Evaluating affected subnetworks and identifying effective drug combinations

When a drug is administered, it will affect disease related genes and cause expression changes at transcription level. Therefore, the weights of disease related genes can reflect the degree of effect to some extent, and are used by our method to define the efficacy of drugs. On the other hand, drug will also affect other “innocent” genes due to the connectivity and complexity of a cellular system. If the affected "innocent" genes are essential genes, side effect may manifest. Therefore, the weights of essential genes in the subnetwork that have no relation with the disease under study are utilized to define the side effect of drugs. Clearly, one good drug should maximize efficacy and minimize side effect, which however are always in conflict and need to be balanced. In this work, the two terms are balanced by a parameter λ, and an evaluation scheme for one drug is formulated as follows:

(14)

where *SD* denotes the set of disease genes in the subnetwork affected by a drug, *BD* is the set of disease genes in the background network, and *ED* denotes the set of essential genes in the subnetwork that have no relation with the disease under study. It can be seen that the first term actually represents efficacy while the second one represents side-effect. Therefore, the balanced score *S_ef f_* can reflect the overall effect of the target drug efficiently. The subnetworks affected by both single drug and combination drug can be evaluated by the score defined above. If the score for a subnetwork affected by certain combination of drugs is higher than that of any member drugs, we conclude that the drug combination is effective.

## Competing interests

The authors declare that they have no competing interests.

## Authors contributions

LNC and XMZ conceived the methods. ZKW conducted the experiments and wrote the manuscript. LNC and XMZ revised the manuscript carefully.

## Supplementary Material

Additional file 1 In this file, enriched GO terms in subnetwork affected by Avandamet were listed. Click here for file

Additional file 2 In this file, enriched GO terms in subnetwork affected by Metformin were listed. Click here for file

Additional file 3 In this file, enriched GO terms in subnetwork affected by Rosiglitazone were listed. Click here for file

Additional file 4 In this file, enriched GO terms in subnetwork affected by Avandamet were listed. Click here for file

Additional file 5 In this file, enriched GO terms in subnetwork affected by Metformin were listed. Click here for file

Additional file 6 In this file, enriched GO terms in subnetwork affected by Rosiglitazone were listed. Click here for file
